# A nationwide, longitudinal collection of patient-reported outcomes from prostate cancer patients and controls

**DOI:** 10.1007/s11136-025-04017-7

**Published:** 2025-07-02

**Authors:** Y. M. Gjelsvik, T. Å. Myklebust, S. D. Fosså, E. S. Haug, R. Kvåle, G. Ursin, T. B. Johannesen

**Affiliations:** 1 Cancer Registry of Norway, Norwegian Institute of Public Health, Oslo, Norway; 2 Department of Research and Innovation, Møre and Romsdal Hospital Trust, Ålesund, Norway; 3 Department of Oncology, Oslo University Hospital, Oslo, Norway; 4 Department of Urology, Vestfold Hospital Trust, Tønsberg, Norway; 5 Department of Clinical Medicine, Bergen University, Bergen, Norway; 6 Department of Oncolocy, Haukeland University Hospital, Bergen, Norway; 7 Lipid Clinic, Oslo University Hospital, Oslo, Norway; 8 Department of Preventive Medicine, Keck School of Medicine, University of Southern California, Los Angeles, CA USA

**Keywords:** Patient-reported outcomes (PROs), Longitudinal health survey, Participation rates, National, Registry-based, Prostate cancer

## Abstract

**Purpose:**

Patient-reported outcomes (PROs) provide valuable information on adverse effects of cancer treatment. The aim of this study was to describe the feasibility of a population-based PROs collection and identify factors affecting participation.

**Methods:**

A total of 13 595 patients diagnosed with prostate cancer (PCa) 2017–2019, along with 10 653 age- and region-matched men with no history of PCa (controls), were invited to a health survey collecting PROs shortly after diagnosis and again one and three years after the initial invitation. Invitations were sent by digital or regular mail. The survey included Norwegian translations of the EQ-5D-5L, EORTC QLQ-C30, and EPIC-26 instruments. We assessed participation rates in all three survey rounds.

**Results:**

We invited 90% of newly diagnosed PCa patients to round 1 of the survey. Participation was higher among patients (58%) than controls (34%), with the highest response rate among digitally invited patients aged 60–69 (66%). Among patients, 44% participated in both rounds 1 and 2. Almost one third (32%) of the invited patients participated in all three rounds. Factors associated with higher participation included digital invitation, higher education, lower age, and being treated with curative intent. Among participants treated with curative intent, 51% completed the first questionnaire before starting local treatment.

**Conclusions:**

National registry-based PROs data collection is feasible for both PCa patients and controls. However, both non-responder and attrition biases are likely to influence questionnaire results. Institutions conducting population-based health surveys should provide information on non-responders and attrition to aid interpretation of questionnaire data.

**Plain English summary:**

The Cancer Registry of Norway invited all Norwegian prostate cancer patients diagnosed between 2017 and 2019 to participate in a three-year health and quality of life survey. This study examines the success of the set-up of this nationwide survey. Patients received the first questionnaire at least six weeks after diagnosis, followed by the second and third questionnaires one and three years later. A control group consisting of men with no history of prostate cancer was also invited. We sent invitations as digital or regular (paper) mail. We used widely recognised questionnaires to assess health and quality of life in prostate cancer patients. Of the 13 595 patients invited, 58% participated in the first round. Among the 10 653 controls invited, 34% participated. The combined participation rate for patients in the first two rounds was 44%, and 32% for all three rounds. We found that digital invitation, higher education, lower age, and curative treatment were associated with higher response rates. We have shown that it is possible to conduct a registry-based national health survey over time among patients and controls. However, questionnaire results may not be representative due to non-responders and responders being different regarding, e.g., education, age and treatment. Therefore, those conducting population-based health surveys should provide information on non-responders and attrition to aid interpretation of questionnaire data.

**Supplementary Information:**

The online version contains supplementary material available at 10.1007/s11136-025-04017-7.

## Introduction

The number of persons living with or after cancer has increased rapidly over the past decades. In Norway, the number of prevalent cases increased by 43% from 2013 to 2023 [1]. Cancer treatment can significantly affect patients’ health-related quality of life (HRQoL) [2], and the burden of cancer treatment on patients may be underestimated [3, 4]. Different clinicians tend to evaluate patients’ symptoms differently [5], and clinicians may describe symptoms as less severe than if expressed directly by patients [6–9], highlighting the importance of directly asking patients [10].

Patient-reported outcomes (PROs) are reported by patients with no intermediate interpretation (e.g., by clinicians) and cover a wide range of issues, from easily observable aspects such as the ability to walk, to less visible concerns like depression or erectile dysfunction [11]. PROs provide valuable insights into patients’ HRQoL, as well as their experience with health services [12, 13]. Self-reported HRQoL measures have been associated with survival in cancer patients [14–17], and completion of electronic PROs questionnaires in clinical practice have been associated with increased survival among patients with advanced cancer [18–20]. One study reported a higher 5-year survival among cancer patients who had completed at least one symptom assessment compared to those who did not [21].

Cancer registries have been used to identify possible participants in several PROs studies [22, 23]. However, as far as we know, the Cancer Registry of Norway (CRN) was first in establishing a national, longitudinal HRQoL survey among both cancer patients and controls, starting shortly after cancer diagnosis [24].

The Norwegian Prostate Cancer Registry is a national registry systematically collecting information on PCa diagnosis and treatment in substantially more detail than a standard cancer registry. Results are used to increase the quality of diagnosis and health care, and therefore, we use the term quality registry throughout the manuscript.

We describe in detail the administration and logistics of the Prostate Cancer Outcomes Norway (PCO-Norway) study, the first PROs collection in a national cancer quality registry in Norway. In this study, we collected PROs data both digitally and with paper forms. The overall aims of PCO-Norway were to acquire experience with collecting population-based PROs and to generate results on HRQoL and adverse effects that could be used to improve PCa care. The prostate cancer (PCa) patient group is heterogenous with considerable variation in age, disease stage, treatment, and symptom burden. These characteristics made the PCa patient group well-suited for a national pilot study on PROs. We analyse our experience with such data collection among PCa patients diagnosed in 2017, 2018 and 2019, along with controls invited during the same period.

Our main aims were to:

- Describe the feasibility of population-based PROs collection among patients and controls conducted by a national cancer registry.
- Identify factors associated with participation.

## Methods

### Study design

Norwegian physicians and healthcare providers are required to report all cancer cases to the CRN. The registry receives data from pathology reports, clinical notifications, death certificates, patient administrative data from the Norwegian Patient Registry, and radiotherapy data from all radiotherapy units [1]. The estimated overall completeness of incident cases in the CRN is 98.8% [25]. The CRN administers the incidence registry for all cancer types as well as nine national quality cancer registries, including the Norwegian Prostate Cancer Registry [26].

Each Norwegian citizen has a unique national identification number, allowing data linkage across sources, as permitted by Norwegian legislation.

PCO-Norway was a population-based, longitudinal cohort study where all men newly diagnosed with PCa between 2017 and 2019 were to be invited to a HRQoL survey starting shortly after diagnosis (preferably before treatment), with follow-up questionnaires after one and three years. As part of this study, participants consented to linkage of survey data with data from the CRN (including the Norwegian Prostate Cancer Registry), the Norwegian Patient Registry, the Medical Birth Registry of Norway, the Norwegian Prescription Database, and Statistics Norway.

PCa patients were identified through the CRN and invited regardless of age, treatment, or disease stage. One of two data sources triggered an invitation to round 1 of the survey for the PCa patients: a pathology report with standardised Norwegian Pathology (NORPAT) codes set by the pathologist confirming PCa, or a clinical notification form. Incomplete or missing pathology coding or delayed clinical notifications regarding PCa cases to the CRN would impact the invitation process. Patients who were deceased or living outside of Norway at the time of inclusion were not invited.

Pathology reports could be received at the registry within days of the biopsy date. To avoid inadvertently informing patients of their diagnosis, a 42-day waiting period was implemented after the biopsy date. However, after consulting with clinicians, we considered the waiting period of 42 days after biopsy to be longer than necessary. Thus, the interval between date of biopsy and invitation was shortened to 21 days in June 2019.

Initially, clinical notifications received in the registry led to an invitation to the survey within 1–6 days based on the assumption that the patient had already been informed about the diagnosis. This assumption was based on the fact that clinical notifications include information on planned treatment, which should already have been discussed with the patient. When we realised that a few patients had been invited based on clinical notifications before being informed about the diagnosis by their doctor, we implemented a waiting period of 21 days after the registry received a clinical notification.

Men residing in Norway and with no previous or current PCa diagnosis (but they could have or have had other cancers) were randomly selected from the National Population Register and invited to the survey as controls. Questionnaire results from the controls would help determine which health issues are common in the age-matched population and which health issues are likely to be associated with PCa at diagnosis and during and after treatment. To reduce postage costs, approximately 8 controls were invited per 10 PCa patients. Potential controls were invited three times per year during the PCO-Norway inclusion period (2017–2019) and were sampled to match the distribution of the invited PCa patients by 10-year age groups (40–49, 50–59, 60–69, 70–79, 80–89, 90–99) and region of residence (southern and eastern, western, central, or northern Norway).

Patients and controls received identical survey invitations titled “Survey on Men’s Health”, to avoid disclosing sensitive personal information in case anyone else than the intended survey recipient saw the invitation letter. Patients received follow-up questionnaires regardless of round 1 participation, while controls only received the follow-up questionnaires if they participated in round 1 (this also helped reduce postage costs).

### Choice of measures

The questionnaire was designed based on recommendations to combine generic instruments with disease-specific instruments [27] and consisted of the Norwegian versions of the EQ-5D-5L [28], the EORTC QLQ-C30 [29], and the EPIC-26 [30, 31], supplemented by one item on sexual interest from the EORTC QLQ-PR25 [32] and specific questions about use and effect of medications/devices for erectile dysfunction [33]. These supplements were in accordance with the International Consortium for Health Outcomes Measurement standard set for localised PCa [34]. The Norwegian translation of EPIC-26 had previously been shown to be valid in curatively treated PCa patients, with acceptable psychometric properties [35, 36]. Norwegian reference data for the EORTC QLQ-C30 had been previously collected [37].

The questionnaire included background questions on relationship status/living situation, education level and height/weight. The one- and three-year follow-up questionnaires included items on treatment and follow-up (only patients were asked these questions), as well as questions on basis of income and work life/work ability [38, 39]. The three-year questionnaire also included items on lymphoedema [32], comorbidities [40], PSA level and use of private health insurance (only patients were asked questions on PSA and private health insurance).

Three items from the Norwegian Cancer Patient Experiences Questionnaire [41] on shared decision making and cooperation between the general practitioner and the hospital, modified to address PCa patients, were included in the one-year follow-up questionnaire (only patients were asked these questions).

### Infrastructure and data collection

**Fig. 1:**
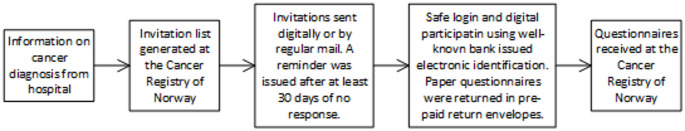
Flowchart of the infrastructure of Prostate Cancer Outcomes Norway

Users of an official digital mailbox (Digipost/eBoks) in the Common Contact Register received invitations digitally. The Norwegian Digitalisation Agency provided the digital mail service. The invitation letter included a survey link, accessible through the official Identification portal (ID-porten), which is a standard safe login solution for Norwegian public services. Participants could use any device with internet access. Invitations were distributed by regular (paper) mail to those who did not have an official digital mailbox and/or had stated a preference against digital communication in the Common Contact Register. These invitations included the questionnaire and a pre-paid return envelope. Paper questionnaires were scanned at the CRN and converted using optical character recognition and manually verified. One reminder was issued after at least 30 days of no response. Figure 1 shows the infrastructure flowchart.

### Feedback and inquiries

Persons with questions regarding the project could call the Cancer Help Line at the Norwegian Cancer Society or contact the CRN. Most inquiries concerned technical issues or reminder letters received even though the person had already participated (this occurred only with paper questionnaires). Between 20 and 23 persons (approx. 4% of all invited) contacted the project each month.

### Statistical methods

We present descriptive statistics for patients and controls as percentages (% invited by digital/regular mail and response rates) and response rates by number of days since diagnosis. To assess differences in participation by education, we described self-reported education level from the analysis sample and contrasted it with summary statistics on education in the general population as reported by Statistics Norway [42]. To investigate how well the participants diagnosed in 2017–2019 represented all PCa patients diagnosed during the same period, we presented distributions of selected medical variables and age at diagnosis for participating patients and all PCa patients. Analyses were done using Stata 17.0 (StataCorp. 2021. *Stata Statistical Software: Release 17.* College Station, TX: StataCorp LLC).

## Results

### Inclusion in PCO-Norway

**Fig. 2:**
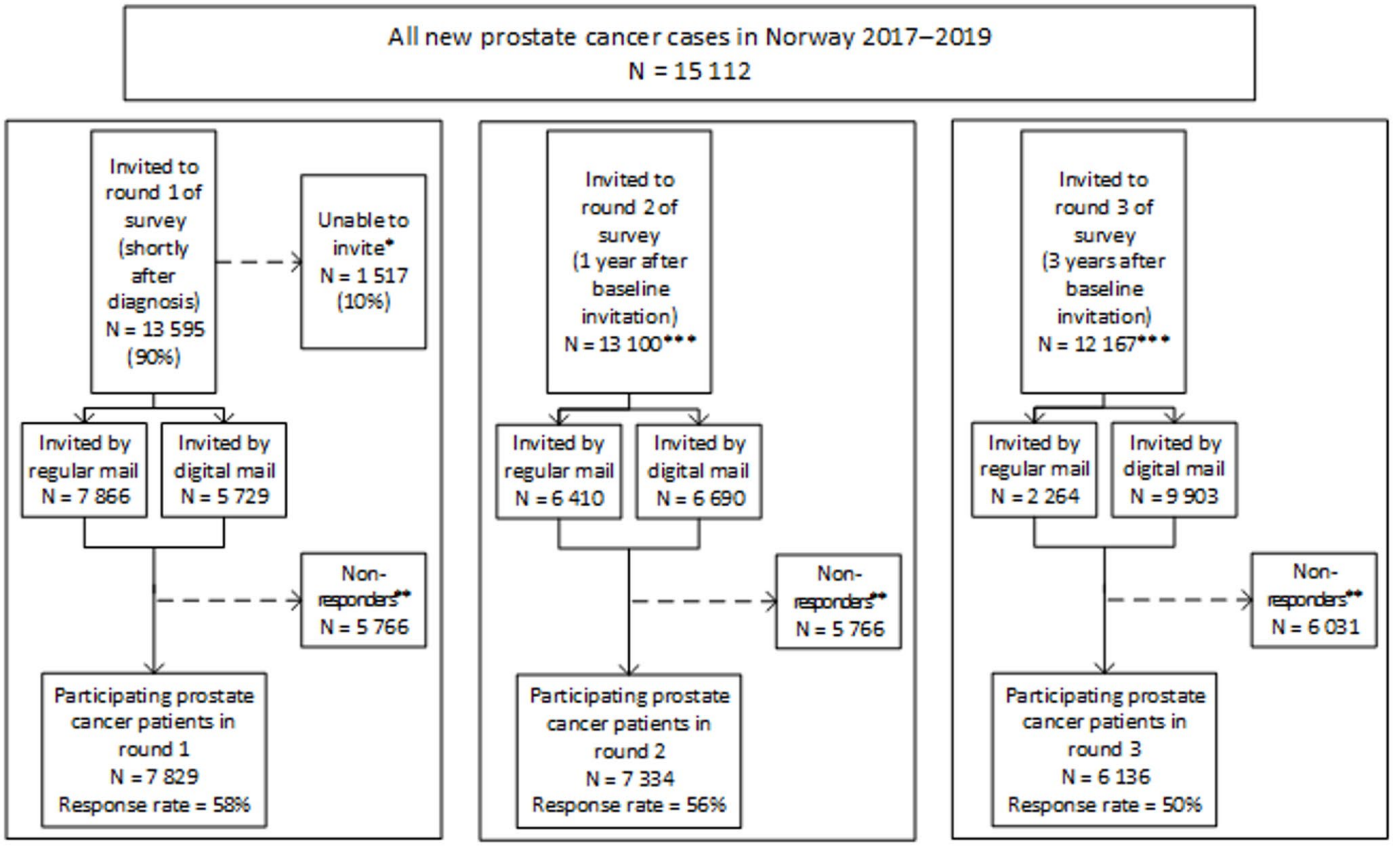
Invitations and responses in all three rounds of Prostate Cancer Outcomes Norway among prostate cancer patients diagnosed in 2017–2019 who were invited to round 1 by 30 June 2020. *Patients not included in the invitation system, main reasons being incomplete/delayed reporting to the Cancer Registry of Norway or patients being deceased at time of study inclusion. **Including men who withdrew their consent ***Men who had passed away, emigrated, or withdrawn their consent since round 1 were excluded for further invitations

Of 15 112 PCa patients registered as diagnosed in 2017–2019 in the CRN (as of 2024), we were able to identify and invite 13 595 (90%) patients until June 2020 (Fig. 2). Based on quality assurance of a sample of 100 of the 1 517 patients who were not invited, the main reasons for non-invitation were incomplete coding of pathology reports in patients who also lacked clinical reports at diagnosis or that patients were deceased or residing abroad. We identified and invited 10 653 potential controls (supplementary Table 2).

Overall, we invited 42% of the patients and 41% of the controls digitally (Fig. 2 and supplementary Tables 1 and 2). The proportion of digital invitations declined with increasing age: only 13% of patients and 10% of controls aged ≥ 85 years received a digital invitation in round 1 (supplementary Tables 1 and 2).

The overall median time between diagnosis and survey participation was 70 days for patients invited by digital mail (diagnosis 2017: 62 days; 2018: 71 days; 2019: 83 days) and 92 days for patients invited by regular mail (diagnosis 2017: 91 days; 2018: 93 days; 2019: 96 days). The increased time from diagnosis to survey participation was mainly due to developmental work on the main database at the CRN during the inclusion period. Notably, the median time from invitation to participation remained relatively stable from 2017 to 2019. Also, some patients diagnosed in late 2019 were not invited to the survey until May 2020, as COVID-19 restrictions led to a pause in invitations from March to May 2020.

### Response rates for round 1 of the survey

**Fig. 3:**
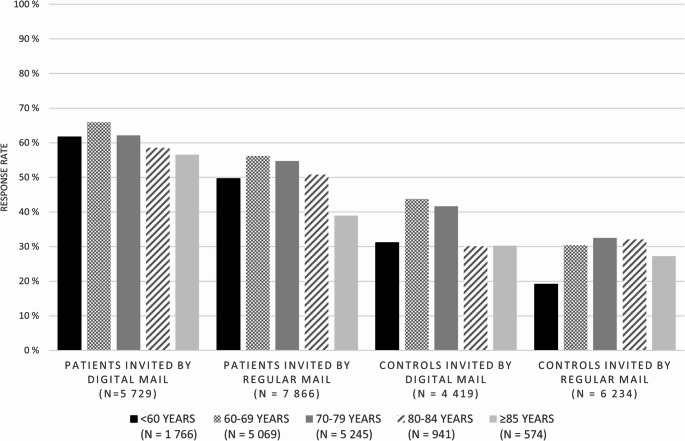
Response rates among prostate cancer patients diagnosed in 2017–2019 and controls invited to round 1 of the survey (*N* = 24 248) grouped by age and invitation method (digital/regular (paper) mail)

In round 1, the overall response rates were 58% for patients invited to participate (*N* = 13 595) and 34% for controls (*N* = 10 653). Response rates were higher among those invited by digital mail (patients: 63%, controls: 40%) compared to those invited by regular mail (patients: 53%, controls: 30%) (supplementary Tables 1 and 2). Additionally, 36% of participants received a reminder before participating in the survey, regardless of invitation method. Response rates among patients were highest (66%) in the 60–69 age group invited digitally and declined with increasing age. A similar pattern emerged among controls (Fig. 3).

### Response rates in follow-up rounds

**Fig. 4:**
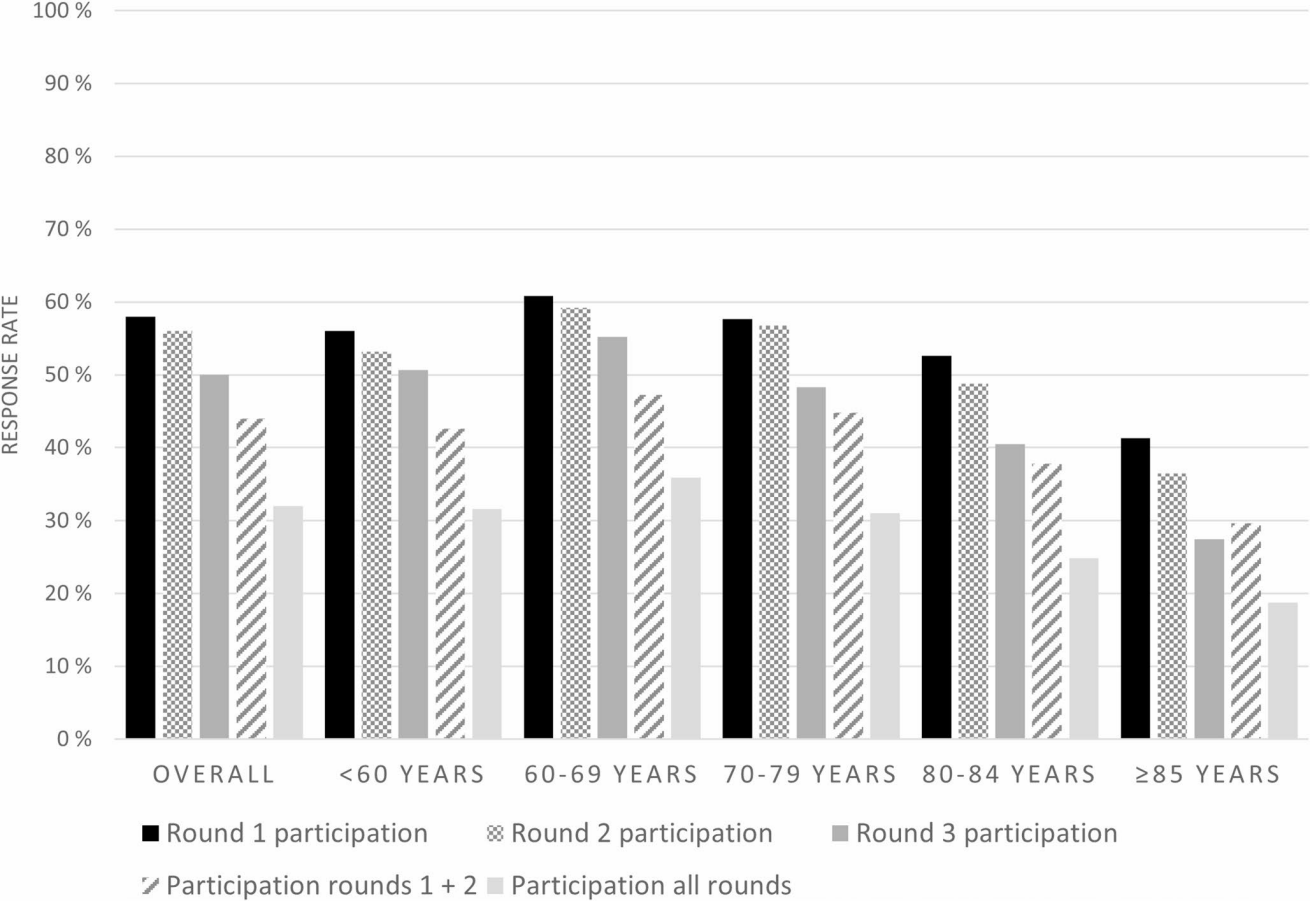
Participation in round 1, round 2, round 3, and participation in the two first/all three rounds among patients diagnosed in 2017–2019 (N invited in round 1 = 13 595, N invited to round 2 = 13 100, N invited to round 3 = 12 167) grouped by age at round 1 invitation. The percentages for participation in round 1 + 2 and all rounds are calculated using the number of patients who were invited to round 2 and 3, respectively, as denominators

Among patients, the overall response rates were 56% in round 2 (one year after the round 1 invitation) and 50% in round 3 (three years after round 1 invitation) (Fig. 2). Overall, 44% of patients participated in both rounds 1 and 2, and 32% of patients participated in all three rounds of the survey. Participation in multiple rounds of the survey declined with increasing age (Fig. 4). Of all round 1 patient participants still alive and residing in Norway one year after the round 1 invitation, 77% participated in round 2 (supplementary Table 3). Among patients participating both in rounds 1 and 2, 71% participated in round 3 (supplementary Table 4).

Only controls who participated in round 1 were invited to rounds 2 and 3 of the survey. The overall response rate for round 2 was 65% (2 318 responders of 3 572 invited). In round 3, 59% of the controls participated (2 005 of 3 416 invited). Of the 3 416 controls invited to round 3, 45% (1 548) participated in all three rounds.

### Pre-treatment participation

**Fig. 5:**
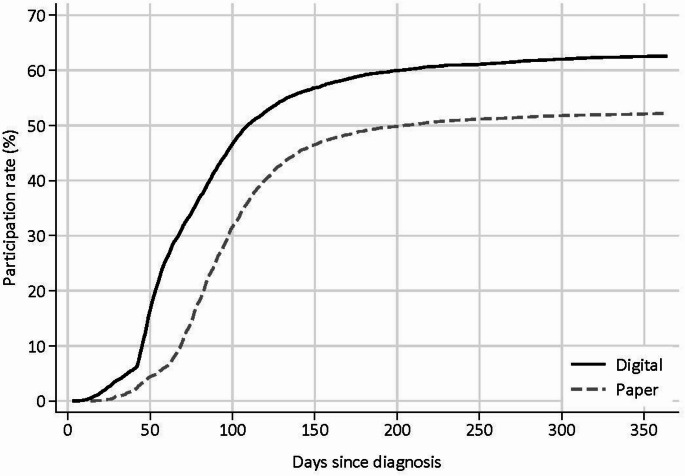
Accumulated response rates in round 1 by number of days since diagnosis for patients diagnosed with prostate cancer in 2017–2019 and invited to the survey (*N* = 13 595). Grouped by invitation method (digital/regular (paper) mail)

Patients and controls invited digitally participated in the survey sooner after diagnosis than those invited by regular mail, likely reflecting the longer delivery time of regular mail. The waiting period between diagnosis and invitation (at least 42 days for most patients) is also reflected in the results (Fig. 5).

Overall, 2 057 of 4 052 (51%) participating patients treated with radical prostatectomy or curative radiotherapy returned the round 1 questionnaire before receiving treatment. Among these, participants who underwent prostatectomy had the lowest proportion of pre-treatment responders, at 32%, while 84% of participants receiving radiotherapy responded before radiation start (hormone therapy status unknown).

### Representativeness of responders

**Fig. 6:**
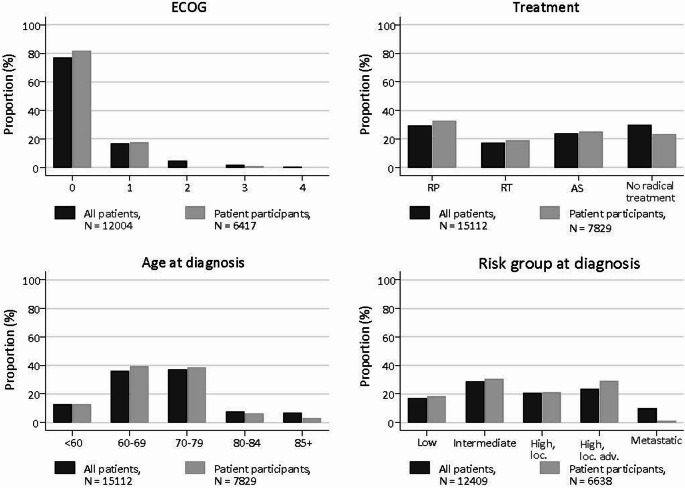
Proportions (%) of Eastern Cooperative Oncology Group (ECOG) functional status: “0 = Fully active, able to carry on all pre-disease performance without restriction, 1 = Restricted in physically strenuous activity but ambulatory and able to carry out work of a light or sedentary nature, e.g., light house work, office work, 2 = Ambulatory and capable of all selfcare but unable to carry out any work activities. Up and about more than 50% of waking hours, 3 = Capable of only limited selfcare, confined to bed or char more than 50% of waking hours, 4 = Completely disabled. Cannot carry on any selfcare. Totally confined to bed or chair.” [43], primary treatment (RP = Radical Prostatectomy, RT = Radiotherapy, AS = Active Surveillance), age group at diagnosis and European Association of Urology risk group [44] (loc. = localised, loc. adv. = locally advanced). All patients registered as diagnosed in 2017–2019 in the Norwegian Prostate Cancer Registry (*N* = 15 112) and all patients participating in round 1 of PCO-Norway (*N* = 7 829)

A higher proportion of PCO-Norway participants (both patients and controls) had a college or university education compared to the general male population in both chosen age groups (supplementary Table 5). Patients who participated were slightly overrepresented in the younger age groups and had somewhat better ECOG performance status [43] than the whole PCa patient group (Fig. 6). Patients who participated were underrepresented in the group with metastatic PCa at diagnosis and in the group that received no radical treatment (probable overlap between the two groups) compared to all patients diagnosed.

### Completeness of returned questionnaires

Questions regarding issues such as sexual function and urinary incontinence were considered some of the most sensitive in the PCO-Norway questionnaire. Overall, more than 90% of the EPIC-26 domains could be analysed according to the official scoring instructions [30]. We observed some variation in EPIC-26 completeness across age groups and response methods; younger patients and digital participants were generally more willing to respond to the EPIC questions (supplementary Table 6).

## Discussion

Our nationwide study successfully invited 90% of newly diagnosed PCa patients. Round 1 survey participation was substantially higher among patients (58%) than controls (34%), and 44% of the patients participated in both round 1 and round 2, one year later. Round 1 participation was highest among digitally invited patients aged 60–69 years (66%) and declined with increasing age. For both patients and controls, participation rates were higher among those invited digitally and those with higher education. Additionally, participating patients were more likely to be treated with curative intent than non-participating patients. Among patients who participated and were treated with radical prostatectomy or curative radiotherapy, 51% returned their questionnaires before the start of local treatment.

To our knowledge, the present study is the first to describe a nationwide, population-based longitudinal PROs collection, initiated and conducted by a cancer registry, including both cancer patients and controls starting shortly after diagnosis. Several HRQoL studies have used data from quality registries in order to identify study participants for planned projects [22, 23]. Population-based collections of self-reported HRQoL among cancer patients and controls have been conducted previously, e.g., the CAESAR + study in Germany [45] and in the PROFILES registry in the Netherlands [46], however little or no data have been collected before treatment [45–48]. In contrast, PCO-Norway was able to collect data for 51% of respondents before treatment with radical prostatectomy or curative radiotherapy. In the SEER-Medical Health Outcomes Survey (MHOS) linkage, 1 930 out of 11 612 cancer patients (17%) had responded to the MHOS both before and after diagnosis in 1998–2001 [49]. In Sweden, hospitals recruit PCa patients to report electronic PROs, with follow-up questionnaires sent by the National Prostate Cancer Register of Sweden [50]. However, not all hospitals recruit patients, and slightly more than 50% of eligible patients received a baseline questionnaire, with just over 30% of the eligible patients submitting a questionnaire before treatment [51]. Recruitment conducted by hospitals, or joint survey recruitment, where the central registry invites all patients not invited by hospitals, has the potential to increase inclusion rates and improve pre-treatment participation. However, as the numbers from Sweden show, this approach requires substantial resources to make it work in practice.

Participation in epidemiological studies has declined since the 1980s [52]. This has been exemplified by declining participation in the HUNT 1–4 studies, large Norwegian regional health studies in which all adults ≥ 20 years in one county were invited. Participation rates were 89% in 1984–86, 70% in 1995–97, 54% in 2006–08, and remained at 54% 2017–19 [53]. In a recent study collecting normative EORTC QLQ-C30 data from the Norwegian general population, the response rate was 33% [54]. Similarly, in public health surveys conducted in three Norwegian counties in 2023, participation was between 29% and 34% [55]. Thus, the round 1 response rates of 58% among the patients and 34% among the controls suggest that participation in PCO-Norway is similar to other contemporary Norwegian population-based health surveys. The decline in response rates in epidemiological studies over the past decades may be a consequence of “survey fatigue” in the population or a general reluctance to comprehensive questionnaires in an environment increasingly shaped by short attention spans and frequent shifts of attention.

While overall response rates were similar in all three rounds of the survey, we observed attrition from round 1 to the follow-up rounds. We also observed participants in rounds 2 and 3 who had not responded in round 1. This may suggest that the timing of the round 1 invitation may have been suboptimal for certain patients e.g., due to ongoing treatment. We also observed lower round 1 participation among patients with metastatic disease.

Overall, 42% of patients included in PCO-Norway were reached by digital mail in round 1. However, the proportion of digitally active persons, also in the older age groups, has increased in the past years [24] and is expected to continue rising. In 2020, the CRN began using the national solution for PROs collection, which made it possible to reach more persons digitally through the official Norwegian health portal, Helsenorge.no. This is reflected in the proportion of patients invited digitally in round 3 (85%) (supplementary Table 4). We observed a higher median number of days between diagnosis and study participation among patients invited by regular mail compared to those invited digitally. Paper questionnaires and invitations had to be printed and sent by regular mail and then received by mail. The paper questionnaires also had to be scanned, optically read and manually verified by a project assistant. When the CRN started collecting PROs data from cancer patients on a regular basis in 2020 [24], this was done using only digital invitations and questionnaires.

Based on the infrastructure and experiences of the PCO-Norway data collection, the CRN now (2025) collects PROs from people with one of 11 cancers, using Norwegian translations of validated generic and disease-specific PROs instruments. Findings are included in the annual reports for the quality registries for these cancers (administered by the CRN), and noteworthy results are brought to the attention of medical leadership.

### Strengths and limitations

The nationwide, population-based design of the study — with fixed time intervals between survey rounds, regardless of factors such as disease stage, treatment, or age — may have lower risk of selection bias compared to clinical PROs studies and provide better representativeness of the PROs data for the PCa patient group as a whole. Although hospital-initiated surveys usually have less attrition, likely due to patients’ sense of loyalty, a nationwide survey reduces the risk of bias due to perceived obligation or gratitude towards the investigator affecting the answers. PCO-Norway data can be used to investigate the quality of Norwegian PCa treatment. Results can be communicated to health care providers and hospital leadership. The choice of PROs instruments in the PCO-Norway questionnaire can facilitate international research cooperation and comparisons on HRQoL in PCa patients.

The moderate round 1 response rate is the most important limitation of PCO-Norway, but this response rate is similar to other health surveys.

A study design where all round 1 questionnaires were completed before treatment was not feasible, as it was essential to ensure that the survey invitation was not sent too early, and specifically not before the patient had been informed about the diagnosis by their clinician. Therefore, approximately half of the participants who received curative treatment had already undergone treatment when they returned their round 1 questionnaire. Treatment experience may therefore have influenced responses from some patients. However, the available questionnaire data from controls can be used to reduce the consequences of this limitation. An alternative approach would be to ask respondents to recall their pre-treatment health status, and this was considered in the planning stage of PCO-Norway. Ultimately, this option was rejected as the risk of recall bias was considered too large.

## Conclusions

We have shown that it is feasible for a national cancer registry to collect nationwide HRQoL data from both patients and controls with reasonable participation rates. However, both non-responder and attrition biases are likely to influence questionnaire results. Institutions conducting population-based health surveys should provide information on non-responders and attrition to aid interpretation of questionnaire data.

## Electronic supplementary material

Below is the link to the electronic supplementary material.

Supplementary Material 1
