# Supplementary material for: A nationwide, longitudinal collection of patient-reported outcomes from prostate cancer patients and controls
Source: Qual Life Res. 2025 Jul 2;34(9):2689–700. doi: 10.1007/s11136-025-04017-7 (PMC12431927; doi:10.1007/s11136-025-04017-7)
Supplement: Supplementary file 1 — Supplementary Material 1 [file 11136_2025_4017_MOESM1_ESM.docx]

# A nationwide, longitudinal collection of patient reported outcomes from prostate cancer patients and controls

Quality of Life Research

YM Gjelsvik^1^, TÅ Myklebust^1,2^, SD Fosså^3^, ES Haug^4,5^, R Kvåle^1,6^, G Ursin^1,7,8^, TB Johannesen^1,3^

*^1^Cancer Registry of Norway, Norwegian Institute of Public Health, Oslo, Norway, ^2^Department of Research and Innovation, Møre and Romsdal Hospital Trust, Ålesund, Norway, ^3^Department of Oncology, Oslo University Hospital, Oslo, Norway, ^4^Department of Urology Vestfold Hospital Trust Tønsberg Norway, ^5^Department of Clinical Medicine, Bergen University, Bergen, Norway, ^6^Department of Oncolocy, Haukeland University Hospital, Bergen, Norway ^7^Lipid Clinic, Oslo University Hospital, Oslo, Norway, ^8^Department of Preventive Medicine, Keck School of Medicine, University of Southern California, Los Angeles, CA, USA*

Corresponding author: Ylva Maria Gjelsvik, [ylva.maria.gjelsvik@fhi.no](mailto:ylva.maria.gjelsvik@fhi.no)

**Table 1** Invitations sent, and responses received in round 1 of the survey among patients diagnosed with prostate cancer in 2017–2019 (N=13 595).

|  | Invitation method | | | | Overall | |
| --- | --- | --- | --- | --- | --- | --- |
|  | Digital mail | | Regular (paper) mail | |  |  |
| Age group | N invited by digital mail (% of all invited) | N responses (response rate) | N invited by regular mail (% of all invited) | N responses (response rate) | N invited by digital or regular mail | N responses (response rate) |
| <60 years | **923 (52%)** | **570 (62%)** | **843 (48%)** | **419 (50%)** | **1766** | **989 (56%)** |
| 60-69 years | **2410 (48%)** | **1588 (66%)** | **2659 (52%)** | **1494 (56%)** | **5069** | **3082 (61%)** |
| 70-79 years | **2098 (40%)** | **1304 (62%)** | **3147 (60%)** | **1722 (55%)** | **5245** | **3026 (58%)** |
| 80-84 years | **222 (24%)** | **130 (59%)** | **719 (76%)** | **365 (51%)** | **941** | **495 (53%)** |
| ≥85 years | **76 (13%)** | **43 (57%)** | **498 (87%)** | **194 (39%)** | **574** | **273 (41%)** |
| Total | **5729 (42%)** | **3635 (63%)** | **7866 (58%)** | **4194 (53%)** | **13595** | **7829 (58%)** |

**Table 2** Invitations sent, and responses received in round 1 of the survey. Control group invited to round 1 of the survey (N=10 653).

|  | Invitation method | | | | Overall | |
| --- | --- | --- | --- | --- | --- | --- |
|  | Digital mail | | Regular (paper) mail | |  |  |
| Age group | N invited by digital mail (% of all invited) | N responses (response rate) | N invited by regular mail (% of all invited)) | N responses (response rate) | N invited by digital or regular mail | N responses (response rate) |
| <60 years | **711 (51%)** | **222 (31%)** | **693 (49%)** | **133 (19%)** | **1404** | **355 (25%)** |
| 60-69 years | **1901 (48%)** | **832 (44%)** | **2054 (52%)** | **624 (30%)** | **3955** | **1456 (37%)** |
| 70-79 years | **1618 (39%)** | **674 (42%)** | **2522 (61%)** | **820 (33%)** | **4140** | **1494 (36%)** |
| 80-84 years | **146 (21%)** | **44 (30%)** | **561 (79%)** | **180 (32%)** | **707** | **224 (32%)** |
| ≥85 years | **43 (10%)** | **13 (30%)** | **404 (90%)** | **110 (27%)** | **447** | **123 (28%)** |
| Total | **4419 (41%)** | **1785 (40%)** | **6234 (59%)** | **1867 (30%)** | **10653** | **3652 (34%)** |

**Table 3** Invitations sent, and responses received in round 2 of the survey among patients diagnosed with prostate cancer in 2017–2019 who participated in round 1 of the survey (N=7 608)

|  | Invitation method | | | | Overall | |
| --- | --- | --- | --- | --- | --- | --- |
|  | Digital mail | | Regular (paper) mail | |  |  |
| Age group at survey baseline | N invited by digital mail (% of all invited) | N responses (response rate) | N invited by regular mail (% of all invited) | N responses (response rate) | N invited by digital or regular mail | N responses (response rate) |
| <60 years | **661 (69%)** | **498 (75%)** | **303 (31%)** | **239 (79%)** | **964** | **737 (76%)** |
| 60-69 years | **1862 (62%)** | **1413 (76%)** | **1158 (38%)** | **933 (81%)** | **3020** | **2346 (78%)** |
| 70-79 years | **1507 (51%)** | **1112 (74%)** | **1439 (49%)** | **1167 (81%)** | **2946** | **2279 (77%)** |
| 80-84 years | **149 (32%)** | **85 (57%)** | **315 (68%)** | **241 (77%)** | **464** | **326 (70%)** |
| ≥85 years | **52 (24%)** | **27 (52%)** | **162 (76%)** | **109 (67%)** | **214** | **136 (64%)** |
| Total | **4231 (56%)** | **3135 (74%)** | **3377 (44%)** | **2689 (80%)** | **7608** | **5824 (77%)** |

**Table 4** Invitations sent, and responses received in round 3 of the survey among patients diagnosed with prostate cancer in 2017–2019 who participated in both round 1 and round 2 of the survey (N=5 591)

|  | Invitation method | | | | Overall | |
| --- | --- | --- | --- | --- | --- | --- |
|  | Digital mail | | Regular (paper) mail | |  |  |
| Age group at survey baseline | N invited by digital mail (% of all invited) | N responses (response rate) | N invited by regular mail (% of all invited) | N responses (response rate) | N invited by digital or regular mail | N responses (response rate) |
| <60 years | **695 (96%)** | **511 (74%)** | **29 (4%)** | **21 (72%)** | **724** | **532 (73%)** |
| 60-69 years | **2106 (92%)** | **1568 (74%)** | **195 (8%)** | **152 (78%)** | **2301** | **1720 (75%)** |
| 70-79 years | **1736 (80%)** | **1136 (65%)** | **424 (20%)** | **331 (78%)** | **2160** | **1467 (68%)** |
| 80-84 years | **172 (59%)** | **91 (53%)** | **121 (41%)** | **82 (68%)** | **293** | **173 (59%)** |
| ≥85 years | **50 (44%)** | **16 (32%)** | **63 (56%)** | **36 (57%)** | **113** | **52 (46%)** |
| Total | **4759 (85%)** | **3322 (70%)** | **832 (15%)** | **622 (75%)** | **5591** | **3944 (71%)** |

**Table 5** Education level in the general Norwegian population and PCO-Norway first round participants ≥40 years (N=11 476). Patients diagnosed 2017–2019. Numbers for the general population was generated using table 08921 from Statistics Norway. <https://www.ssb.no/statbank/table/08921>

| Age group | Highest education level | Patient participants (N=7 829) | Control participants (N=3 652) | Norwegian male population aged ≥40 years, 2017 (N=1 295 912) |
| --- | --- | --- | --- | --- |
| 40–59 | Primary school | **9 %** | **10 %** | **20 %** |
|  | Secondary school | **46 %** | **40 %** | **47 %** |
|  | College/University | **44 %** | **51 %** | **33 %** |
|  | Unknown | **1 %** | **0 %** | **1 %** |
| 60+ | Primary school | **18 %** | **19 %** | **22 %** |
|  | Secondary school | **38 %** | **36 %** | **50 %** |
|  | College/University | **42 %** | **44 %** | **27 %** |
|  | Unknown | **1 %** | **1 %** | **1 %** |

**Table 6** Completeness of EPIC-26 domains among prostate cancer patients participating in round 1 of PCO-Norway (N=7 829)

| Invitation method | EPIC domain | Age group | | | | |
| --- | --- | --- | --- | --- | --- | --- |
|  |  | <60 | 60–69 | 70–79 | 80–84 | ≥85 |
| Digital mail | Urinary incontinence | **99%** | **97%** | **96%** | **90%** | **88%** |
|  | Urinary obstructive | **98%** | **96%** | **93%** | **91%** | **84%** |
|  | Bowel | **99%** | **98%** | **96%** | **94%** | **93%** |
|  | Sexual | **94%** | **92%** | **90%** | **83%** | **81%** |
|  | Hormonal | **100%** | **99%** | **99%** | **95%** | **95%** |
| Regular mail | Urinary incontinence | **98%** | **96%** | **93%** | **91%** | **84%** |
|  | Urinary obstructive | **97%** | **94%** | **90%** | **86%** | **78%** |
|  | Bowel | **99%** | **96%** | **92%** | **88%** | **83%** |
|  | Sexual | **98%** | **97%** | **96%** | **93%** | **85%** |
|  | Hormonal | **96%** | **95%** | **91%** | **88%** | **80%** |
